# Factors associated with unhealthy behaviours and health outcomes: a cross-sectional study among tuscan adolescents (Italy)

**DOI:** 10.1186/s12939-014-0083-5

**Published:** 2014-09-25

**Authors:** Giacomo Lazzeri, Elena Azzolini, Andrea Pammolli, Rita Simi, Veronica Meoni, Mariano Vincenzo Giacchi

**Affiliations:** CREPS-Research for Health Education and Promotion, University of Siena Italy, Siena, Italy; Department of Molecular and Developmental Medicine, University of Siena Italy, Via A. Moro 2, 53100 Siena, Italia; Department of Public Health, Catholic University of the Sacred Heart, Roma, Italy; Local Health Unit 7, Siena, Italy

**Keywords:** Inequalities in health, Peers, School environment, FAS, Nutritional status

## Abstract

**Background:**

We aimed to determine the extent to which three core variables (school environment, peer group and family affluence) were associated with unhealthy behaviours and health outcomes among Tuscan adolescents. The unhealthy behaviours considered were smoking, alcohol consumption, sedentary lifestyle and irregular breakfast consumption; health outcomes were classified as self-reported health, multiple health complaints and life satisfaction. School environment was measured in terms of liking school, school pressure, academic achievement and classmate support; peer groups were evaluated in terms of the number of peers and frequency of peer contact. Family affluence was measured on a socioeconomic scale.

**Methods:**

Data were taken from the Tuscan 2009/10 survey of “Health Behaviour in School-aged Children”, a WHO cross-national survey. A binary logistic multiple regression (95% confidence intervals) was implemented.

**Results:**

The total sample comprised 3291 school students: 1135 11-year-olds, 1255 13-year-olds and 901 15-year-olds. Peer group and school environment were associated with unhealthy behaviours such as smoking, alcohol consumption and sedentary lifestyle. Family affluence proved to have less impact on unhealthy behaviours, except in the case of adolescents living in low-income families. Poor health outcomes were directly related to a negative school environment. Regarding the influence of family affluence, the results showed higher odds of life dissatisfaction and poor self-reported health status in medium-income families, while low-income families had higher odds only with regard to life dissatisfaction. A consistent pattern of gender differences was found in terms of both unhealthy behaviours and health outcomes.

**Conclusions:**

Unhealthy behaviours are strongly related to the school environment and peer group. A negative school environment proved to have the strongest relation with poor health outcomes.

## Introduction

One of the main priorities of European public health decision-makers is to reduce health inequalities, which persist in spite of the “Health for All” policy of the World Health Organization (WHO) [1]. According to Health 2020 the European policy for health and well-being, it is of primary importance to address the social determinants of health and to reduce health inequalities. Health is influenced by the way in which people live, their access to health care, schools and leisure opportunities, their homes, communities and towns. Although socioeconomic inequalities are known to influence health-related behaviour, little is known about the differential effects of health promotion across socioeconomic groups. Several studies have correlated unhealthy behaviours, such as physical inactivity, unhealthy eating habits, smoking and alcohol consumption, with lower socioeconomic status [2-6]. However, other studies have found weak or non-existent relationships between socioeconomic status (SES) and health behaviours [7-13].

Among adolescents, unhealthy behaviour in the school environment has been associated with low academic achievement, obesity, poor self-reported health status, more numerous health complaints, regular smoking, longer time spent watching TV, unhealthy eating habits and drunkenness, and poor emotional well-being, life-skills, health behaviours and life satisfaction [10,11,14,15]. Some studies have found that smoking and physical inactivity are associated with the size of the peer group, and that smoking, alcohol use and physical inactivity are connected with the frequency of peer contact [16,17]. Various authors have reported that health inequalities related to school and peer environments are found both in risky health behaviours, such as smoking and alcohol consumption, and in various positive health behaviours [18]. Moreover, studies on socioeconomic status have found that differences among youths are better explained by the school environment and peer group rather than by SES [10,15,16].

The main aim of the present study was to ascertain which of the following factors – school environment, peer group, family affluence (an indicator of SES as measured by Family Affluence Scale (FAS)), gender, municipality size and nutritional status – were associated with unhealthy behaviours and negative health outcomes in Tuscan adolescents. A further aim was to determine the relative magnitude of these factors, in order to identify the primary influences on health behaviours and health outcomes within the study group.

## Materials and methods

### Study

Data were taken from the Tuscan 2009/10 survey of “Health Behaviour in School-aged Children” (HBSC), a WHO cross-national survey which collects data every fourth year from a random sample of schools [19,20]. The Ethics Committee of the National Institute of Health approved the protocol and agreed to the use of an opt-out consent form.

### Design, sampling and data collection

The methods used to gather these data are described in detail elsewhere [19]. An anonymous structured questionnaire was administered in classrooms by trained personnel [20]. Dependent and independent variables were considered in the analysis.

### Dependent variables

Five specific measures of unhealthy behaviour (smoking, alcohol consumption, physical inactivity, sedentary lifestyle and irregular breakfast consumption) and three measures of health outcomes (multiple health complaints, self-reported health and life satisfaction) were used as dependent variables.

Adolescent smoking habits were assessed by asking the participants how often they smoked tobacco. Response options were “every day”, “at least once a week”, “less than once a week” and “I don’t smoke”. Subsequently, smokers (the first three response categories) were compared with non-smokers.

The level of sedentary lifestyle was measured by asking participants how many hours a day they spent watching television or using a computer, play-station or similar media devices [21,22]. The use of screen-based media (SBM) was scored by summing the mean number of hours per day engaged in screen-based activities. Respondents’ behaviour was regarded as positive when they spent no more than two hours a day in front of the TV or PC screen.

Physical activity (PA) was defined as “any activity that raises your heart rate and which possibly leaves you out of breath”. Respondents’ behaviour was regarded as negative if they did not meet the physical activity guideline (PAGL) (at least 60 minutes of PA seven days a week). Participants were categorized as “not meeting PAGL” or “meeting PAGL” [23].

In order to assess the frequency of breakfast consumption during the week (Monday to Friday), respondents were asked to indicate how many days a week they had breakfast. Having breakfast five days a week was considered to be a positive health behaviour, while less frequent breakfasting was classed as “irregular breakfast habits”.

Alcohol use was assessed by the question: “How often do you drink alcohol, such as beer or wine?”. Responses were registered on a five-point scale. Infrequent drinking (rarely or never) was regarded as healthy behaviour, while other patterns were classed as unhealthy behaviour.

Respondents were regarded as suffering from multiple health complaints if they reported experiencing two or more symptoms “more than once a week” or “about every day” [24]. Respondents were assessed for eight symptoms on a five-point scale: difficulty in falling asleep, headache, feeling dizzy, stomach-ache, backache, depression, irritability or bad temper, and nervousness [25].

Self-reported health was assessed by means of a four-point scale; “fair” or “poor” perceptions were classed as “poor self-reported health” [19].

General life satisfaction was assessed by means of the Cantril ladder (1–10 points) [19]. Participants were shown a picture of a ladder and asked: “The top of the ladder (10) is the best possible life for you and the bottom (1) is the worst possible life. In general, where on the ladder do you feel you stand at the moment?”. A score of 5 or less was taken to indicate dissatisfaction [19].

### Independent variables

The independent variables used in the analysis were: family affluence, school environment, peer group, nutritional status and demographic size of the adolescent’s municipality of residence. These analyses were controlled for gender, as gender differences have been reported in the literature [26,27].

Socioeconomic status was evaluated by means of the FAS, Currie et al. have reported the scale’s characteristics and modality of use [19].

Principal-component analysis was used to calculate a one-dimensional representation of the school environment. The analysis considered six variables, three concerning scholastic activity – “feeling pressured by schoolwork (retrospectively recorded)”, “academic achievement”, “liking school” – and three concerning peer support – “students in my class like being together”, “students in my class are kind and helpful” and “students in my class accept me for who I am”. On summing the number of indicators, the resulting first major component corresponded to 33% of the overall variance. Saturations of individual variables ranged from 0.36, for “academic achievement”, to 0.68, for “students in my class are kind and helpful”. The new composite variable “school environment” was mainly representative of the three items on peer support, and considerably less so for the other three items (“liking school”, “feeling pressured by schoolwork” and “academic achievement”). Lastly, the scores derived from the first component factor were recorded in a new categorical variable, “school environment”, consisting of three categories: “favourable”, “medium” and “poor”.

The peer group indicator was used as a one-dimensional indicator which took into account both the frequency of peer contact and the size of the peer group. While the number of factors was limited to one, there were originally four variables: “time spent after school with friends”, “number of close friends of the opposite gender”, “electronic communications with friends” and “number of close friends of the same gender”. From the total variance, 38% was assigned to the first main component extracted. The new composite variable “peer group” was mainly influenced by the size of the peer group (number of close friends), while “contact with peers” (electronically and after school) had less impact. Individual variables displayed saturations within a range of 0.30-0.80, where the lower end reflected “electronic communications” and the higher end reflected “number of close friends of the opposite gender”. Moreover, the scores derived from the first component factor were recorded in a new categorical variable, “peer group”, consisting of three categories: “favourable”, “medium” and “poor”.

### Nutritional status

Self-reported weight and height were used to calculate Body Mass Index (BMI in kg/m^2^). We applied age- and gender- specific cut-offs, as recommended by the International Obesity Task Force [28,29]. Both underweight (U) and normal-weight (N) subjects were grouped into the “Under/Normal-weight” (UN) category, while both overweight (Ow) and obese (O) individuals constituted the “Overweight group” (Ow/O).

### Demographic size of the adolescent’s municipality of residence

In order to determine the demographic size of the adolescents’ municipalities of residence, the samples were divided into four categories:<10,000 inhabitants; 10,000-50,000 inhabitants; >50,000 inhabitants, and >50,000 within a metropolitan area, according to the National Statistics Institute classification [30].

### Statistical analysis

Analysis was carried out by means of the SPSS 20.0 statistical software package (SPSS Inc., Chicago, IL, USA). Binary logistic regression analysis was used to produce adjusted odds ratios (ORs) with 95% CIs and asymptotic, two-sided, statistical significance. Throughout this paper, statistical significance is defined by the conventional levels of *P* < 0.05 and *P* < 0.01.

## Results

Data were obtained from a sample of 3,291 school students, 1,135 of whom were 11-year-olds (34.5%); 1,255 were 13-year-olds (38.1%), and 901 were 15-year-olds (27.4%).

The sample showed a high prevalence of unhealthy behaviours. Indeed, 22.0% of the students smoked, 84.7% used SBM for more than two hours a day, 91.6% reported physical inactivity and 34.5% skipped breakfast on weekdays (Table 1). Moreover, 42.1% had multiple health complaints and 13.3% stated that they were dissatisfied with their lives (Table 1).

**Table 1:** **Unhealthy behaviours and health outcomes among Tuscan adolescents**

	**N**	**%**
**Smoking**		
Non-smokers	2551	(78.0)
Smokers	720	(22.0)
Total	3271	(100.0)
**Screen-based media use**		
<2 hours a day	504	(15.3)
≥2 hours a day	2783	(84.7)
Total	3287	(100.0)
**Physical activity**		
≥ 60 min 7 days a week	275	(8.4)
Less often	2982	(91.6)
Total	3257	(100.0)
**Breakfast on weekdays**		
Five days a week	2126	(65.5)
Less often	1121	(34.5)
Total	3247	(100.0)
**Alcohol consumption**		
Never or rarely	2475	(76.2)
Every day	774	(23.8)
Total	3249	(100.0)
**Multiple health complaints**		
< 2 complaints more than once a week	1899	(57.9)
≥ 2 complaints more than once a week	1381	(42.1)
Total	3280	(100.0)
**Self-rated health**		
Excellent or good	2957	(90.1)
Fair or poor	324	(9.9)
Total	3281	(100.0)
**Life satisfaction**		
Satisfied	2837	(86.7)
Dissatisfied	437	(13.3)
Total	3274	(100.0)

Table 2 shows the independent variables. A total of 39.8% of respondents reported medium family affluence; 50.0% described their school environment as medium and 50.1% described their peer group as medium (Table 2).

**Table 2:** **Independent variables**

**Composite variables**	***n*** **(%)**	**Total (100%)**	**Original variables**	***n*** **(%)**	**Total (100%)**
**Family affluence**			Family car		
High	1689 (52.5)	3217	None	104 (3.2)	3271
Medium	1279 (39.8)		One	1044 (31.9)	
Low	249 (7.7)		Two or more	2123 (64.9)	
			Own bedroom		
			No	1130 (34.7)	3253
			Yes	2123 (65.3)	
			Holiday with family		
			Not at all	298 (9.1)	3261
			Once	905 (27.8)	
			Two or more times	1058 (63.1)	
			No. of computers		
			None	55 (1.7)	3267
			One	1155 (35.4)	
			Two or more	2057 (62.9)	
**School environment**			Students like being together		
Favourable	809 (25.0)	3231	Agree	2796 (85.3)	3276
Medium	1615 (50.0)		Undecided or disagree	480 (14.7)	
Poor	807 (25.0)		Students kind and helpful		
			Agree	2093 (64.0)	3272
			Undecided or disagree	1179 (36.0)	
			Accepted by students		
			Agree	2499 (76.5)	3267
			Undecided or disagree	768 (23.5)	
			Liking school		
			A lot	2094 63.9)	3279
			Less than a lot	1185 (36.1)	
			Academic achievement		
			Good or very good	1789 (54.7)	3269
			Average or below	1480 (45.3)	
			Pressured by schoolwork		
			Not pressured	345 (10.5)	3277
			Pressured	2932 (89.5)	
**Peer group**			Friends same gender		
Favourable	793 (24.8)	3195	3 or more	2488 (75.9)	3279
Medium	1600 (50.1)		Up to 2	791 (24.1)	
Low	802 (25.1)		Friends different gender		
			3 or more	1791 (55.3)	3241
			Up to 2	1450 (44.7)	
			After school with friends		
			4 or more days a week	1266 (38.9)	3257
			3 or fewer days a week	1991 (61.1)	
			Electronic communication		
			Every day	1857 (43.2)	3272
			Less often	1415 (56.8)	
Non-composite variables					
**Municipality size**			**Gender**		
Metropolitan	806 (24.5)	3291	Male		1702 (51.7) 3291
<10,000	456 (13.9)		Female		1589 (48.3)
10,000-50,000	797 (24.2)		**Nutritional status**		
>50,000	1232 (37.4)		Underweight		53 (2.0) 2640
			Normal weight		2192 (83.0)
			Overweight		342 (13.0)
			Obese	53 (2.0)	

### Differences in unhealthy behaviours

Table 3 shows the results of multiple logistic models to associate unhealthy behaviours by age. On comparing high family affluence with medium and low family affluence, it emerged that the latter two associated with lower odds of regular drinking at age 15. Higher odds of irregular breakfast consumption at age 13 were associated with a low FAS. FAS was not significantly linked to any other negative health behaviour. Compared with a “favourable” school environment, “medium” and “poor” environments were associated with significantly higher odds of current smoking and irregular breakfasting at age 13. Moreover, the odds of smoking at age 15 and alcohol consumption at ages 13 and 15 were higher within “poor” school environments. On comparing a favourable peer group with medium and poor peer groups, the latter two proved to be associated with a significantly lower likelihood of smoking and sedentary lifestyle at age 13. “Poor” peer groups showed lower odds of leading a sedentary lifestyle at age 11, and with smoking and alcohol consumption at age 15. The data also displayed a gender difference, in that females drank alcohol less frequently than their male counterparts at 11 and 13 years of age, and tended to have more irregular breakfast habits at ages 13 and 15. Females also proved to be less sedentary, but more prone to smoking, than males at age 15. On comparing overweight (Ow/O) with normal-weight subjects, the odds of physical inactivity were significantly higher at age 15. In terms of geographic location, associations were found only with regard to 11-year-olds; municipalities with less than 10,000 inhabitants and those with 10,000–50,000 inhabitants were associated with lower odds of physical inactivity, while metropolitan municipalities were associated with lower odds for alcohol abuse (Table 3).

**Table 3:** **Adjusted odds ratios with 95% CI: association of gender, family affluence, nutritional status, municipality size, school environment and peer group with unhealthy behaviours, by age**

	**Smoking**	**Sedentary**	**Not meeting PAGL**	**Irregular breakfast**	**Alcohol daily**
**Age 11**					
**Family affluence**					
Medium vs. High	0.42 (0.14–1.27)	0.76 (0.53–1.07)	1.05 (0.61–1.79)	1.00 (0.69–1.47)	1.27 (0.68–2.37)
Low vs. High	0.28 (0.03–2.40)	0.61 (0.35–1.08)	1.30 (0.48–3.55)	1.12 (0.60–2.09)	-
**School environment**					
Medium vs. Favourable	2.47 (0.63–9.83)	1.19 (0.83–1.70)	0.67 (0.39–1.17)	1.44 (0.96–2.18)	1.00 (0.50–1.98)
Poor vs. Favourable	4.33 (0.97–19.3)	1.50 (0.91–2.46)	1.44 (0.61–3.43)	1.65 (0.99–2.77)	0.97 (0.39–2.40)
**Peer groups**					
Medium vs. Favourable	0.18 (0.05–0.64)**	0.74 (0.47–1.14)	1.43 (0.78–2.59)	0.51 (0.32–0.79)**	0.57 (0.26–1.26)
Poor vs. Favourable	0.58 (0.18–1.89)	0.54 (0.33–0.91)*	1.40 (0.65–2.98)	1.12 (0.68–1.84)	1.58 (0.68–3.67)
**Gender**					
Female vs. Male	0.71 (0.25–2.02)	0.82 (0.59–1.14)	1.75 (1.03–2.98)**	1.22 (0.86–1.75)	0.15 (0.06–0.36)**
**Nutritional status**					
Ow/O vs. UN	2.25 (0.72–6.99)	1.51 (0.95–2.41)	1.44 (0.68–3.05)	1.18 (0.75–1.87)	1.12 (0.52–2.39)
**Municipality size**					
Metropolitan vs. >50,000	0.95 (0.20–4.46)	0.93 (0.61–1.43)	0.53 (0.26–1.07)	0.81 (0.51–1.28)	0.31 (0.12–0.79)**
<10,000 vs. >50,000	2.56 (0.65–10.0)	0.81 (0.51–1.30)	0.43 (0.21–0.89)*	1.04 (0.64–1.71)	0.77 (0.34–1.70)
10,000-50,000 vs. >50,000	2.20 (0.55–8.76)	0.77 (0.49–1.21)	0.42 (0.20–0.89)*	0.70 (0.42–1.16)	0.56 (0.23–1.40)
**Age 13**					
**Family affluence**					
Medium vs. High	0.70 (0.48–1.02)	0.79 (0.49–1.27)	1.08 (0.61–1.89)	1.00 (0.76–1.34)	0.71 (0.50–1.01)
Low vs. High	0.43 (0.14–1.25)	0.57 (0.22–1.46)	0.61 (0.20–1.84)	2.17 (1.12–4.19)*	0.66 (0.28–1.56)
**School environment**					
Medium vs. Favourable	2.37 (1.37–4.10)**	1.20 (0.68–2.12)	1.04 (0.55–1.99)	1.65 (1.15–2.37)**	1.55 (0.99–2.41)
Poor vs. Favourable	4.34 (2.41–7.80)**	1.44 (0.73–2.84)	1.13 (0.52–2.44)	2.04 (1.35–3.09)**	1.79 (1.08–2.96)*
**Peer groups**					
Medium vs. Favourable	0.45 (0.30–0.67)**	0.29 (0.13–0.66)**	1.48 (0.81–2.69)	0.97 (0.69–1.34)	0.57 (0.39-0.84)**
Poor vs. Favourable	0.48 (0.30–0.78)**	0.15 (0.06–0.35)**	1.62 (0.77–3.40)	0.90 (0.61–1.32)	0.91 (0.59–1.41)
**Gender**					
Female vs. Male	1.16 (0.81–1.66)	0.72 (0.45–1.15)	1.27 (0.75–2.16)	1.59 (1.20–2.10)**	0.71 (0.51–0.99)*
**Nutritional status**					
Ow/O vs. UN	1.18 (0.73–1.92)	1.37 (0.67–2.79)	2.50 (0.88–7.07)	1.38 (0.94–2.02)	1.31 (0.85–2.03)
**Municipalities size**					
Metropolitan vs. >50,000	1.27 (0.80–2.0)	1.28 (0.71–2.30)	0.96 (0.48–1.90)	0.93 (0.65–1.33)	1.04 (0.67–1.60)
<10,000 vs. >50,000	1.10 (0.66–1.83)	1.09 (0.57–2.08)	2.82 (0.94–8.44)	0.76 (0.50–1.14)	1.04 (0.65–1.69)
10,000-50,000 vs. >50,000	0.90 (0.55–1.46)	1.84 (0.97–3.48)	0.66 (0.35–1.25)	1.19 (0.83–1.71)	1.22 (0.80–1.87)
**Age 15**					
**Family affluence**					
Medium vs. High	0.88 (0.65–1.19)	0.98 (0.56–1.71)	1.26 (0.74–2.15)	0.93 (0.68–1.26)	0.71 (0.52–0.96)*
Low vs. High	0.92 (0.50–1.67)	0.91 (0.33–2.52)	5.0 (0.66–37.6)	1.80 (0.98–3.31)	0.54 (0.29–0.99)*
**School environment**					
Medium vs. Favourable	1.31 (0.87–1.97)	0.58 (0.25–1.34)	0.89 (0.45–1.79)	0.74 (0.49–1.13)	1.22 (0.81–1.85)
Poor vs. Favourable	1.66 (1.07–2.59)*	0.79 (0.32–1.98)	1.27 (0.58–2.81)	1.15 (0.74–1.80)	1.66 (1.06–2.58)*
**Peer groups**					
Medium vs. Favourable	0.87 (0.61–1.23)	1.05 (0.55–2.03)	1.53 (0.85–2.74)	1.29 (0.90–1.83)	0.74 (0.52–1.04)
Poor vs. Favourable	0.62 (0.42–0.93)*	1.08 (0.52–2.23)	1.26 (0.65–2.45)	1.00 (0.67–1.49)	0.53 (0.36–0.79)**
**Gender**					
Female vs. Male	1.49 (1.11–1.99)**	0.45 (0.26–0.80)**	2.05 (1.20–3.50)**	1.65 (1.23–2.22)**	0.82 (0.62–1.10)
**Nutritional status**					
Ow/O vs. UN	1.02 (0.66–1.57)	2.77 (0.84–9.11)	5.59 (1.34–23.4)*	1.42 (0.92–2.19)	0.76 (0.49–1.17)
**Municipality size**					
Metropolitan vs. >50.000	1.02 (0.71–1.48)	0.59 (0.30–1.18)	0.99 (0.52–1.88)	0.87 (0.59–1.26)	1.36 (0.94–1.97)
<10,000 vs. >50,000	0.89 (0.43–1.83)	0.94 (0.20–4.29)	1.04 (0.29–3.73)	0.72 (0.34–1.52)	0.88 (0.43–1.82)
10,000-50,000 vs. >50,000	1.21 (0.86–1.70)	0.59 (0.31–1.12)	0.99 (0.54–1.82)	0.97 (0.68–1.37)	1.13 (0.80–1.60)

Multivariate binary logistic regression *p < 0.05; **p < 0.01.

Ow/O: Overweight group (overweight and obesity); UN: Under/Normal-weight group.

### Differences in health outcomes

In comparison with high-income families, medium-income families displayed higher odds of poor self-reported health at age 15 and lower life satisfaction at age 11. In low-income families, dissatisfaction with life was greater in the 13- and 15-year age-groups. With regard to school environments, 11- and 13-year-old respondents from “medium” and “poor” environments displayed higher odds of poor health outcomes on all three health measures than those from “favourable” environments. Moreover, at age 15, the odds of poor self-reported health and life dissatisfaction were higher in “poor” school environments. Health outcomes did not display peer group influence. Gender seemed to be a determining factor in poor self-reported health, with higher odds among male participants of all ages. However, females reported more health complaints at ages 13 and 15 and greater life dissatisfaction at age 13 than their male counterparts. Comparison between overweight and normal-weight participants revealed higher odds of poor self-reported health at ages 13 and 15 among the overweight. With regard to the demographic size of the municipalities of residence, smaller municipalities displayed higher odds of poor self-reported health at age 13 (Table 4).

**Table 4:** **Adjusted odds ratios with 95% CI: association of gender, family affluence, nutritional status, municipality size, school environment and peer group with health outcomes, by age**

	**Multiple health complaints**	**Poor self-reported health**	**Life dissatisfaction**
**Age 11**			
**Family affluence**			
Medium vs. High	1.24 (0.90–1.70)	0.72 (0.37–1.40)	1.78 (1.06–2.98)*
Low vs. High	1.33 (0.78–2.26)	1.64 (0.66–4.09)	1.73 (0.77–3.86)
**School environment**			
Medium vs. Favourable	1.37 (0.98–1.93)	3.0 (1.30–6.93)**	2.13 (1.16–3.92)**
Poor vs. Favourable	1.90 (1.22–2.95)**	4.29 (1.67–11.0)**	4.24 (2.14–8.38)**
**Peer group**			
Medium vs. Favourable	0.95 (0.64–1.40)	1.58 (0.63–4.0)	1.14 (0.59–2.21)
Poor vs. Favourable	1.29 (0.81–2.03)	1.60 (0.58–4.40)	1.33 (0.64–2.76)
**Gender**			
Female vs. Male	1.24 (0.92–1.68)	2.91 (1.52–5.58)**	1.21 (0.75–1.96)
**Nutritional status**			
Ow/O vs. UN	0.95 (0.63–1.41)	1.57 (0.77–3.19)	1.21 (0.68–2.14)
**Municipality size**			
Metropolitan vs. >50,000	0.83 (0.56–1.23)	0.66 (0.28–1.54)	0.52 (0.27–1.00)
<10,000 vs. >50,000	1.40 (0.92–2.14)	1.05 (0.44–2.51)	1.02 (0.54–1.92)
10,000-50,000 vs. >50,000	1.12 (0.74–1.72)	1.18 (0.53–2.62)	0.63 (0.32–1.23)
**Age 13**			
**Family affluence**			
Medium vs. High	0.95 (0.71–1.26)	0.79 (0.48–1.29)	1.26 (0.83–1.92)
Low vs. High	0.98 (0.50–1.91)	1.87 (0.75–4.70)	3.34 (1.53–7.31)**
**School environment**			
Medium vs. Favourable	2.38 (1.65–3.45)**	3.86 (1.49–10.03)**	3.15 (1.51–6.56)**
Poor vs. Favourable	4.46 (2.91–6.82)**	9.56 (3.66–25.02)**	7.63 (3.60–16.15)**
**Peer group**			
Medium vs. Favourable	0.98 (0.70–1.36)	0.93 (0.51–1.69)	1.26 (0.73–2.15)
Poor vs. Favourable	0.96 (0.65–1.42)	1.18 (0.63–2.23)	1.59 (0.90–2.83)
**Gender**			
Female vs. Male	2.37 (1.79–3.13)**	2.17 (1.33–3.53)**	3.18 (2.04–4.96)**
**Nutritional status**			
Ow/O vs. UN	1.39 (0.94–2.04)	1.83 (1.03–3.26)*	1.24 (0.71–2.17)
**Municipality size**			
Metropolitan vs. >50,000	1.07 (0.74–1.53)	1.04 (0.51–2.09)	1.51 (0.88–2.57)
<10,000 vs. >50,000	0.87 (0.58–1.30)	1.99 (1.02–3.91)*	1.34 (0.73–2.45)
10,000-50,000 vs. >50,000	0.89 (0.62–1.28)	2.13 (1.16–3.92)**	1.28 (0.74–2.21)
**Age 15**			
**Family affluence**			
Medium vs. High	0.90 (0.66–1.24)	1.62 (1.04–2.52)*	1.11 (0.72–1.17)
Low vs. High	0.99 (0.54–1.84)	0.92 (0.36–2.37)	2.41 (1.18–4.95)**
**School environment**			
Medium vs. Favourable	0.87 (0.56–1.33)	1.45 (0.68–3.12)	0.91 (0.44–1.86)
Poor vs. Favourable	1.56 (0.98–2.47)	2.72 (1.26–5.87)**	3.43 (1.72–6.85)**
**Peer group**			
Medium vs. Favourable	1.29 (0.90–1.84)	0.79 (0.646–1.35)	0.97 (0.57–1.64)
Poor vs. Favourable	1.17 (0.78–1.76)	1.01 (0.57–1.78)	1.54 (0.89–2.66)
**Gender**			
Female vs. male	3.43 (2.54–4.65)**	2.50 (1.57–3.96)**	1.41 (0.92–2.15)
**Nutritional status**			
Ow/O vs. UN	1.47 (0.94–2.31)	2.81 (1.63–4.83)**	1.51 (0.86–2.65)
**Municipality size**			
Metropolitan vs. >50,000	1.08 (0.74–1.59)	1.33 (0.75–2.37)	1.08 (0.56–1.85)
<10,000 vs. >50,000	1.18 (0.56–2.51)	1.59 (0.54–4.64)	1.11 (0.36–3.42)
10,000-50,000 vs. >50,000	1.11 (0.77–1.58)	1.66 (1.00–2.76)	1.00 (0.61–1.63)

Multivariate binary logistic regression *p < 0.05; **p < 0.01.

Ow/O: Overweight group (overweight and obesity); UN: Under/Normal-weight group.

## Discussion

This study examined the impact of some of the most prominent factors influencing the health of adolescents, i.e. socioeconomic status, school environment and peer group. Inequalities exerting a direct impact on unhealthy behaviour were found to be associated more with the “peer group” and “school environment”, than with SES. Unhealthy behaviours were associated with a negative school environment, a finding which confirmed the initial hypothesis. Similarly, health outcomes proved to be more closely linked to “school surroundings” than to “peer group” and “family affluence”. With regard to health outcomes, the findings only partially supported the initial hypothesis, as we observed a correlation with the school environment, but not with the peer group.

### Differences in unhealthy behaviours

Previous studies have described the impact of school environment and peer group influence on unhealthy behaviours among adolescents [10,11]. Unlike the findings of some studies [7,18], our results suggest that the role of the peer group and the school environment is more prominent than that of family affluence in determining risky health behaviours such as smoking and alcohol abuse. As age increases, the influence of the family on adolescents declines, while that of peers increases. In accordance with the literature [31], we found that being overweight was associated with low levels of physical activity (not meeting PAGL), especially among 15-year-olds, and with high values of poor self-reported health at 13 and 15 years of age [32]. Again in agreement with the literature, we also observed that living in smaller municipalities was associated with a higher risk of failing to meet the physical activity guideline (at 11 years of age) and of having poor self-reported health (at 13 years) [32].

### Inequalities in health outcomes

Socioeconomic conditions, school environment and peer group all seem to play a role in causing inequalities in health. However, while all three measures of poor health outcomes were directly related to a negative school environment, they displayed no correlation with the peer group. This result is in line with previous research. The findings of previous studies [19,33] were also confirmed on comparing high- and low-income families, in that the latter were associated with higher odds of poor self-reported health and life dissatisfaction. In contrast with previous reports [24,34], however, our findings did not reveal an apparent socioeconomic gradient in multiple health complaints. From our study, it emerged that boys enjoyed a considerable advantage over girls in terms of health status, which confirms the results reported in the literature [26,35]. Nevertheless, among both males and females, both unhealthy behaviours and poor health status were associated with a poor school environment.

An important limitation of our study is that we could not ascertain parents’ habits, which are known to be very important in shaping children’s personal identity [36] and health habits [37,38]. Furthermore, it should be borne in mind that the data collected in this survey were self-reported by participants, and that self-reporting may introduce some errors which could influence the statistical relationships. This suggests that the actual relationships between the variables considered in the study might be distorted. However, the large sample size and the fairly consistent trend in results across the various municipalities suggest that the effects observed are solid. Nevertheless, further studies on other samples will be needed in order to confirm and generalise these results.

Our findings highlight the need for a wide-ranging strategy of intervention in low-income categories. Such intervention should focus both on reducing socioeconomic disparities in adolescents’ health and on improving students’ social position within the peer group and school entourage.

It is essential to involve schools in the design of programmes to promote healthy lifestyles. The main objective of health promotion in schools should not simply be to draw up a curriculum that promotes healthy choices, but rather to organise coherent pedagogical practices that promote critical thinking, a sense of belonging, self-esteem and the feeling of being part of a supportive society, thereby helping adolescents to acquire the skills needed to act in the community.

## Abbreviations

WHO: World Health Organization
SES: Socioeconomic Status
FAS: Family Affluence Scale
HBSC: Health Behaviour in School-aged Children
SBM: Screen-Based Media
PA: Physical Activity
PAGL: Physical Activity Guideline
BMI: Body Mass Index
U: Underweight
N: Normal-weight
Ow: Overweight
O: Obesity
Ow/O: Overweight including Obesity
